# Therapy optimization in multiple sclerosis: a prospective observational study of therapy compliance and outcomes

**DOI:** 10.1186/1471-2377-14-49

**Published:** 2014-03-13

**Authors:** Patricia K Coyle, Bruce A Cohen, Thomas Leist, Clyde Markowitz, MerriKay Oleen-Burkey, Marc Schwartz, Mark J Tullman, Howard Zwibel

**Affiliations:** 1 Department of Neurology, Stony Brook University, Health Sciences Center T12-020, Stony Brook, NY 11794-8121, USA; 2 Department of Neurology, Northwestern University, 710 North Lake Shore Drive, Abbott Hall 1121, Chicago, IL 60611, USA; 3 Comprehensive MS Center, Thomas Jefferson University, 900 Walnut Street, #200, Philadelphia, PA 19107, USA; 4 Multiple Sclerosis Center at Penn, University of Pennsylvania, 3400 W. Spruce St., 3 Gates Bldg, Philadelphia, PA 19104, USA; 5 Health Outcomes Research Consulting and Writing, Outcomes Scribe, LLC, 664 Wynding Oaks, Kalamazoo, MI 49006, USA; 6 Biostatistics, MedNet Solutions, Inc, 110 Cheshire Lane, Suite 300, Minnetonka, MN 55305, USA; 7 The MS Center for Innovations in Care, Missouri Baptist Medical Center, 3009 North Ballas Road, Bldg B, Suite 207B, St. Louis, MO 63131, USA; 8 Neuroscience Consultants, Comprehensive Multiple Sclerosis Center, 4601 Ponce de Leon Blvd., Suite 100, Coral Gables, FL 33146, USA

**Keywords:** Multiple sclerosis, Disease-modifying therapy, Compliance, Relapses, Disability, Quality of life, Work productivity

## Abstract

**Background:**

Data sources for MS research are numerous but rarely provide an objective measure of drug therapy compliance coupled with patient-reported health outcomes. The objective of this paper is to describe the methods and baseline characteristics of the Therapy Optimization in MS (TOP MS) study designed to investigate the relationship between disease-modifying therapy compliance and health outcomes.

**Methods:**

TOP MS was designed as a prospective, observational, nationwide patient-focused study using an internet portal for data entry. The protocol was reviewed and approved by Sterling IRB. The study was registered with ClinicalTrials.gov. It captured structured survey data monthly from MS patients recruited by specialty pharmacies. Data collection included the clinical characteristics of MS such as MS relapses. Disability, quality of life and work productivity and activity impairment were assessed quarterly with well-validated scales. When events like severe fatigue or new or worsening depression were reported, feedback was provided to treating physicians. The therapy compliance measure was derived from pharmacy drug shipment records uploaded to the study database. The data presented in this paper use descriptive statistics.

**Results:**

The TOP MS Study enrolled 2966 participants receiving their disease-modifying therapy (DMT) from specialty pharmacies. The mean age of the sample was 49 years, 80.4% were female, 89.9% were Caucasian and 55.7% were employed full or part time. Mean time since first symptoms was 11.5 years; mean duration since diagnosis was 9.5 years. Patient-reported EDSS was 3.5; 72.2% had a relapsing-remitting disease course. The most commonly reported symptoms at the time of enrollment were fatigue (74.7%), impaired coordination or balance (61.8%) and numbness and tingling (61.2%). Half of the sample was using glatiramer acetate and half was using beta-interferons.

**Conclusion:**

Demographic and clinical characteristics of the TOP MS sample at enrollment are consistent with other community-based MS samples, and the sample appears to be representative of DMT users in the US. TOP MS data can be used to explore the associations between disease-modifying therapy compliance and health outcomes.

**Trial registration:**

ClinicalTrials.gov (NCT00819000)

## Background

Multiple Sclerosis (MS) is one of the most common diseases of the central nervous system [1]. The socioeconomic burden of MS begins with the onset of symptoms most typically in the third and fourth decade of life and extends over many years. Although there is no cure for MS, the disease-modifying therapies (DMTs) have been shown to reduce relapses and slow the progression of disability [1-3].

A core issue for treatment of MS is medication compliance, defined as the extent to which a patient acts in accordance with the prescribed interval and dose of a dosing regimen [4]. Drug compliance is important to ensure patients receive the maximum benefit from their treatment and minimize worsening of their disease. Examining the relationship between DMT compliance and health outcomes in MS was the rationale for designing and implementing the Therapy Optimization in Multiple Sclerosis (TOP MS) study.

Several data sources have been used to examine health outcomes in MS [5-8]. The NARCOMS Registry consists of a large database of MS patients who are followed semi-annually with self-administered questionnaires including validated patient-reported outcome measures [5]. The New York State MS Consortium Registry offers physician-reported outcomes for its participants [6]. Both data sources rely on patient self-reports about DMT compliance [5,6]. Databases of healthcare claims from managed care plans or a claims database from a Pharmacy Benefit Management company with a specialty component offer prescription claims for estimates of drug therapy compliance and a reliable algorithm-based MS relapse measure, but they contain no data on disability, duration of disease, or quality of life [7-9].

To meet the needs of the TOP MS study for both drug compliance and MS health outcomes, US specialty pharmacies that provide DMTs for MS patients were invited to assist with study recruitment. Web-based surveys to capture health outcomes would supplement their medication therapy management (MTM) programs for MS designed to optimize treatment [10]. The pharmacies were willing to provide DMT shipment information to the TOP MS study database that could be used to calculate the compliance measure they most commonly use, the Medication Possession Ratio (MPR) [4,11].

The objectives of this report are to describe the methods and baseline characteristics of the TOP MS study.

## Methods

The TOP MS study used a prospective, open-label parallel group study design involving approximately 3000 persons with MS who were receiving immunomodulatory therapy from one of three specialty pharmacies: Diplomat Specialty Pharmacy, BioScrip Inc., and Walgreens Specialty Pharmacy. The objective of TOP MS was to determine the association of varying levels of compliance with MS therapy on patient outcomes. The study was sponsored and conducted by Teva Pharmaceuticals, Inc, with oversight by the TOP MS Study Steering Committee, a group of neurologists who specialize in the treatment and management of MS.

Inclusion criteria for TOP MS included males and females, 18 years of age or older, with a diagnosis of MS, who were continuing or initiating treatment with approved doses of glatiramer acetate (GA, Copaxone, Teva Pharmaceuticals, Inc.), intramuscular or subcutaneous interferon beta-1a (IFNβ-1a IM; Avonex, Biogen Idec; IFNβ-1a SC, Rebif, EMD-Serono), or interferon beta-1b (IFNβ-1b, Betaseron, Bayer Pharmaceuticals, Inc.). No restrictions were placed on the number of enrolled patients using each of the DMTs.

Exclusion criteria were limited to contraindications to the immunomodulators (including pregnancy, trying to become pregnant or breastfeeding), and any condition that might interfere with participation for the full 24-month duration of the study.

There were no disallowed previous medications. Concomitant medications could include any treatments for MS except investigational drugs. Any concomitant diseases were allowed and recorded.

The study protocol was reviewed and approved by Sterling IRB. The TOP MS study was registered with ClinicalTrials.gov (NCT00819000).

MedNet Solutions, a database development company, was contracted to build a research-quality database that could capture the patient-reported data for the study. MedNet also hosted the web portal.

Before beginning study procedures, pharmacy study managers received training from the study sponsor on MS and study procedures. Thereafter, they participated in weekly teleconferences to discuss study issues.

Prior to study enrollment, treating physicians with potential participants from each specialty pharmacy received an informational brochure or letter about the TOP MS study. They were invited to receive biannual participant-specific reports during the study suitable for inclusion in the patients’ charts.

When patient recruitment began, study managers called potential participants to introduce the TOP MS study. If patients agreed to receive additional information, IRB-approved informed consent forms (ICF) outlining the procedures and evaluations for the study were mailed. Signed ICF were returned to the pharmacies, and study managers initiated study enrollments through the web-based portal. Demographic characteristics for all potential participants and the enrolled sample were available from the pharmacies and are presented in Table 1.

**Table 1:** Characteristics of persons contacted, excluded, lost, and enrolled

**Characteristic**	**Contacted, no response**	**Refused participation**	**Enrolled, lost before baseline**	**Enrolled successfully**
	**(n = 6766) n%**	**(n = 3710) n%**	**(n = 185) n%**	**(n = 2966) n%**
**Age (Years)**				
≤ 30	536 ( 7.9)	164 ( 4.4)	7 ( 3.8)	140 ( 4.7)
31 – 40	1434 (21.2)	517 (13.9)	30 (16.3)	455 (15.3)
41 – 50	2207 (32.6)	1000 (27.0)	54 (29.2)	885 (29.8)
51 – 60	1873 (27.7)	1291 (34.8)	65 (34.9)	1068 (36.0)
≥ 61	716 (10.6)	738 (19.9)	29 (15.8)	418 (14.1)
**Gender**				
Female	5200 (76.9)	2815 (75.9)	153 (82.8)	2385 (80.4)
Male	1566 (23.1)	895 (24.1)	32 (17.2)	581 (19.6)
**Dispensed DMT:**				
GA	3187 (47.1)	1677 (45.2)	99 (53.6)	1475 (49.7)
IFNβ-1a IM	1504 (22.2)	857 (23.1)	30 (16.3)	604 (20.4)
IFNβ-1a SC	1172 (17.3)	678 (18.3)	29 (15.8)	470 (15.8)
IFNβ-1b	903 (13.3)	498 (13.4)	27 (14.4)	417 (14.1)

*DMT*, Disease-modifying therapy; *IFNβ-1a IM*, Intramuscular interferon beta-1a; *IFNβ-1b*, Interferon beta -1b; *GA*, Glatiramer acetate; *IFNβ-1a SC*, Subcutaneous interferon beta-1a.

The enrollment process produced electronic log-on instructions for the portal. A link to technical support was also available. Beginning at baseline and at regular intervals over 24 months, enrolled participants received electronic messages to respond to survey questions within two-week windows. Near the end of the windows telephone reminders were initiated. Self-reported responses were entered directly into the study database and stored for later analyses. Those who changed or stopped DMT and were willing to continue to provide responses to questionnaires were allowed to remain in the study. If three consecutive months of surveys were missed, and participants could not be reached by telephone, they were considered to have discontinued the study.

The therapy compliance measure, MPR, was derived from pharmacy shipment records. MPR was defined as the ratio of days that the patient had drug to take at the prescribed frequency to the number of days in the interval (i.e., 12 months), expressed as a decimal or percentage [11,12].

### Data collection

Study questionnaires for the clinical characteristics of MS were designed by the TOP MS Study Steering Committee and included MS history, comorbidities, MS therapy history, relapses (history and presence at baseline), and targeted adverse events. Table 2 presents the modules captured at each study time point.

**Table 2:** Data collection by time points

**Modules**	**Baseline**	**Months 1,2,4,5,7,8, 10,11,13,14 16,17,19,20, 22,23**	**Months 3,9,15,21**	**Months 6, & 18**	**Months 12 & 24**
**Demographic characteristics**	X				
**Co-morbidities baseline/updates**	X		X	X	X
**MS history**	X				
**MS therapies & concomitant drug history/updates**	X		X	X	X
**Relapses, history and current**	X				
**Current DMT**		X	X	X	X
**Current MS status (relapse)**		X^f^	X^f^	X^f^	X
**Targeted events**^**a**^	X	X	X	X	X
**Quality of Life (SF-12v.2™)**	X		X	X	X
**Disability: Self-Assessed Kurtzke**	X			X	X
**Fatigue Severity Scale (FSS)**	X		X	X	X
**Patient Health Questionnaire (PHQ-9)**^**b**^	X		X	X	X
**Perceived Deficits Questionnaire (PDQ-5)**^**c**^	X			X	X
**Work Productivity and Activity Impairment: General Health (WPAI:GH)**	X		X	X	X
**Drug shipment data for compliance & persistence**^**d**^	X	X	X	X	X
**Termination**^**e**^					X
					24 months

*DMT*, Disease-modifying therapy; *SF-12v.2™*, Short Form Health Survey, 12 items, version 2, [14].

^a^Most commonly reported adverse events; Includes reports of pregnancy; ^b^Depression screener/severity assessment [17,18]; ^c^Cognition screener [19]; ^d^Shipment data were uploaded electronically to study database; ^e^Termination module was asked when a patient left the study or completed follow-up. ^f^At report of relapse, Self-Assessed Kurtzke was completed and repeated in six months [13].

Disability was assessed with the validated Self-Assessed Kurtzke developed by Dr. L.C. Scheinberg. A self-report disability rating scale, the responses are highly correlated with scores on the Expanded Disability Status Scale (EDSS) [13].

Health-related quality of life was assessed with the validated Short Form Health Survey, 12 items, version 2 (SF-12v.2^®^) that generates Physical and Mental Component Scores [14]. Our database was designed to allow completed SF-12v.2 forms to be scored by QualityMetric, the scale developer. They submitted scores directly to the TOP MS Study database.

For those employed full or part time, the validated Work Productivity and Activity Impairment: General Health (WPAI:GH) scale was used to assess lost work time due to MS and the impact of MS on completing tasks while at work [15]. All participants completed the scale question concerning the interference of MS with doing usual daily activities other than employment.

Covariates used in the study included MS disease type, disease duration, demographic characteristics, comorbidities, fatigue, depression and cognitive deficits.

MS disease type was determined from responses to specific questions on the TOP MS survey dealing with relapse history, onset of initial symptoms, and subject assessment of their MS relative to six months prior used as a proxy for progression. Disease duration was measured 1) from time of first symptoms that the physician attributed to MS and 2) from date of diagnosis.

Comorbidities were queried at baseline and updated every three months. Fatigue severity was assessed with the validated 9-item Fatigue Severity Scale (FSS) every three months [16]. The Patient Health Questionnaire (PHQ-9) was used to screen for depression (first two questions), and the total score for the nine items determined severity [17,18]. Cognitive deficits were assessed every six months with the validated 5-item Perceived Deficits Questionnaire (PDQ-5) developed specifically for MS patients [19]. The study included faxed or mailed follow-up with treating physicians when events such as severe fatigue or new depression or worsening depression were reported.

Safety assessment in the TOP MS Study included monthly queries about commonly reported adverse events associated with the study DMTs. Serious Adverse Events (SAEs) meeting the criteria of seriousness for regulatory requirements were reviewed by the study Medical Monitor for relatedness to study DMTs and submitted to manufacturers’ Pharmacovigilance departments. Reported pregnancies were also tracked.

### Data analysis

After data collection was completed, the biostatistician extracted the study data from the study website database server, and the data were analyzed using “R” software, version 3.0.0 or higher (The R Foundation for Statistical Computing, Vienna, Austria). Data for each module were maintained in separate data structures, linked by unique participant identifiers. At no time during the study did the study sponsor have access to patient names or contact information.

In this report, we present frequency distributions for selected variables from the baseline survey modules.

## Results

### Evaluating representativeness

The enrolled TOP MS sample (n = 2966) was evaluated for representativeness relative to those who were contacted about participation but did not respond (n = 6766), those who refused to participate (n = 3710), and those who enrolled but did not complete the baseline survey and were lost from the study (n = 185). We present the comparative data in Table 1 as benchmarks. While the enrolled sample was somewhat more likely to be female, more likely to be 51 to 60 years old and less likely to be 40 years or younger, the distribution of DMT use was similar to the full sample of potential participants. There was nationwide representation with participants and nonparticipants from all fifty states, District of Columbia and Puerto Rico.

### Demographic characteristics at baseline

Table 3 describes the demographic characteristics of the enrolled TOP MS sample.

**Table 3:** Baseline demographic characteristics of prevalent MS patients

**Characteristic**	**Mean**	**SD**
**Age** (Years)	49.0	10.3
**Characteristic**	**n**	**%**
**Race:**		
Caucasian	2666	89.9
Black/African American	172	5.8
Hispanic/Latino	68	2.3
Mixed race	31	1.0
Other races	29	1.0
**Gender:** Female	2385	80.4
**Employment status:**		
Employed, full time	1294	43.6
Employed, part time	290	9.8
Employed at Home	69	2.3
Disabled due to MS	697	23.5
Retired	251	8.5
Homemaker	190	6.4
Unemployed	142	4.8
Student	30	1.0
Workers’ compensation	3	0.1
**Positive smoking status:**	517	17.4

*SD*, Standard Deviation.

The mean age was 49 years (Median: 50 years; Range: 18 to 78 years). The sample was predominantly Caucasian and female, and 55.7% were in the labor force, working full or part time. The prevalence of smoking in this enrolled sample was 17.4%; however, those who were disabled due to MS or were unemployed for other reasons had significantly higher rates of smoking than those who were employed or retired.

### Disease characteristics: duration, activity, symptoms and disability status

Disease characteristics are detailed in Table 4. While the mean time since first symptoms was 11.5 years, the median time was 9.3 years with a range of less than one month to 60 years. The mean age at first symptoms was 37.5 years ± 10.2 years. The mean time since diagnosis was 9.5 years with a median time of 7.4 years and a range of less than one year to 47 years. More than 80% reported they were diagnosed by physician assessment accompanied by magnetic resonance imaging (MRI). About 75% were being treated by an MS specialist neurologist. Nearly three-quarters of the enrolled sample were judged to have relapsing-remitting MS based on their responses to pre-defined questions.

**Table 4:** Baseline disease characteristics of prevalent MS patients

**Characteristic**	**Mean**	**SD**
**Time since first symptoms** (years)	11.5	9.5
**Time since diagnosis** (years)	9.5	8.3
**Characteristic**	**n**	**%**
**Disease course:**^**a**^		
Relapsing-remitting	2136	72.2
Secondary progressive	351	11.9
Primary progressive	437	14.8
Progressive-relapsing	26	0.9
Clinically Isolated Syndrome	8	0.3
**Number of patient-reported relapses (not physician confirmed) in past year:**		
0	1455	49.1
1	652	22.0
2	314	10.6
3	130	4.4
4	52	1.8
>4	110	3.7
Uncertain	253	8.5
**Current patient-reported symptoms:**^**b**^		
Fatigue	2215	74.7
Impaired coordination or balance	1834	61.8
Numbness and tingling	1814	61.2
Problems with thinking or memory	1599	53.9
Decreased strength in arms or legs	1548	52.2
Difficulty walking or moving legs	1442	48.6
Bladder problems	1350	45.5
Spasticity	1262	42.5
Pain	895	30.2
Visual symptoms	889	30.0
Bowel problems	774	26.1
Sexual dysfunction	734	24.7
Difficulty with speech or swallowing	637	21.5
Difficulty moving arms or hands	621	20.9
Tremor	527	17.8
Other^c^	239	8.1
**Disease-modifying therapy:**		
GA	1475	49.7
IFNβ-1a IM	604	20.4
IFNβ-1a SC	470	15.8
IFNβ-1b	417	14.1

*SD*, Standard Deviation; *GA*, Glaitramer acetate; *IFN*, Interferon; *IM*, Intramuscular; *SC*, Subcutaneous.

^a^Derived from responses to questions about confirmed relapses, onset of initial symptoms, and MS status relative to six months ago; 8 subjects did not have required information. ^b^Multiple responses were allowed; ^c^Most commonly: Severe headaches/migraines, vertigo/dizziness, ringing in the ears, hearing loss, seizures.

Disability level as measured with the Self-Assessed Kurtzke was a median of 3.5 with an Inter-Quartile Range of 0.0 to 5.5 and a mean of 3.2 (range: 0 to 8.5).

About half of the participants reported having no relapses in the year prior to enrollment. When asked about physician-confirmed relapses, 17.2% reported one confirmed relapse in the prior year while 6.3% reported two confirmed relapses. The percentage of subjects reporting one and two relapses irrespective of physician confirmation were 22.0% and 10.6%, respectively. MRI was reported in conjunction with symptoms of relapses by 18.8%, and 21.7% received corticosteroid treatment in the year prior to enrollment.

When asked about first MS symptoms and additional symptoms they had experienced, participants reported that many of them had persisted and were present at study enrollment. The most commonly reported symptoms at the time of enrollment were fatigue (74.7%), impaired coordination or balance (61.8%) and numbness and tingling (61.2%).

As a requirement of study enrollment everyone was continuing or initiating treatment with a DMT. There were no significant differences in gender, race or current symptoms across the DMT groups.

### Disease characteristics: psychosocial parameters

Table 5 presents the baseline psychosocial parameters. The mean baseline scores on the Physical and Mental Component Scores of the SF-12.v2^®^ were below the 50th percentiles compared to the US population [14]. These component scores reflected the respective subscale scores: the lowest mean scores and poorest quality of life assessment were found on Physical Function (mean and standard deviation [SD]: 40.9 ± 13.2) and Role Physical (mean and SD: 41.2 ± 11.9) subscales while the highest scores came on Bodily Pain (mean and SD: 46.6 ± 12.1) and Mental Health (mean and SD: 47.4 ± 10.7) subscales..

**Table 5:** Baseline psychosocial characteristics of prevalent MS patients

**Characteristic**	**Mean**	**SD**
**Health-related quality of life component scores:**^**a**^		
Physical component score	42.6	11.8
Mental component score	46.9	11.2
**Fatigue:**^**b**^		
FSS score	38.1	15.8
**Depression:**^**c**^		
PHQ-9 score	6.6	5.7
**Work productivity & activity impairment:**^**d**^		
Absenteeism (%)	3.8	14.9
Presenteeism (%)	14.4	20.4
Overall productivity loss (%)	15.8	22.1
Usual activity impairment (%)	35.0	30.8

*SD*, Standard Deviation.

^a^Ware, JE et al. SF-12^®^ Health Survey [14].

^b^Krupp LB et al. The Fatigue Severity Scale (FSS) [16].

^c^Kroenke K et al. The Patient Health Question, 9 items (PHQ-9) [17,18].

^d^Reilly MC, et al. Work Productivity and Activity Impairment-General Health (WPAI-GH) [15]

The mean FSS score at baseline was 38.1 points, and 56.4% of participants met the criteria for severe fatigue with mean baseline scores of 36 points or more.

Overall, 55.2% of participants screened positively for major depression with scores of ≥ 3 points on the first two questions of the PHQ-9. The mean baseline score for the full nine items for all participants was 6.6 points on a range of 0 to 27 suggesting mildly severe depression.

Of those who were employed and responded to the WPAI-GH, 1055 reported missing 3.8% of their normal work hours due to their MS in the past 7 days (absenteeism). Additionally, 14.4% reported that while working they were limited in the amount or kind of work they could do (presenteeism). Combining the two forms of work productivity loss showed that there was an overall productivity loss of 15.8% over the past 7 days. There was a 35.0% impairment due to MS in regular non-employment activities such as work around the house, shopping, childcare, exercising or studying.

## Discussion

The TOP MS Study was designed to provide a patient-focused assessment of the effect varying levels of DMT compliance could have on MS-related disease outcomes. The enrolled sample appeared to be representative of the entire sample of MS patients available for recruitment from the three US specialty pharmacies.

The database produced from the study is unique in capturing DMT compliance from DMT shipment data and does not rely solely on patient self-reports. Concordance of self-reports and other measures of drug compliance have been shown to vary widely, and self-reported compliance is often significantly overestimated due to patients’ desires to provide socially acceptable responses [20-22]. Bruce et al. recently conducted a longitudinal study to compare compliance outcomes for patients with MS using retrospective self-reports, adherence diaries, and an electronic monitoring device that captured time and date of needle disposals [22]. While all measures of compliance were correlated, patients reported better compliance than was shown in diaries or devices. These authors concluded that patient self-reports alone would underestimate poor DMT compliance [22]. A somewhat more objective measure of compliance than self-reports, the use of prescription refill data to calculate MPR, had been used in retrospective claims database research in MS [7]. The use of prescription refill data reported as shipment data from pharmacies is expected to provide a more accurate estimate of compliance than self-reports for TOP MS.

The health outcomes for TOP MS are self-reported by patients. Such self-reported surveys can provide reliable patient-derived disability and clinical data as well as assessments of health-related quality of life [23,24]. Investigations comparing electronically collected patient-reported outcomes to conventional pencil-and-paper collection have concluded that data collected electronically are valid and of comparable quality [24,25].

Key clinical characteristics suggest that the TOP MS sample is representative of other community-based prevalence samples of MS patients. Although somewhat younger at a mean age of 49.0 years compared to the NARCOMS Registry 2008 Spring sample with a mean age of 53.4 years, the years from diagnosis were correspondingly less for the TOP MS sample at a mean of 9.5 years relative to the NARCOMS sample at 14.8 years. Both samples were predominantly Caucasian and female [5]. The Clinformatics™ for Data Mart sample from 2006 to 2010 was also more than 80% female but had a somewhat lower mean age of 45.2 years [7].

Compared to large observational studies of MS such as the Sonya Slifka Longitudinal Multiple Sclerosis Study in the US which looked at natural history of MS and The Global Adherence Project (GAP) conducted in twenty-two other countries, TOP MS had comparable proportions of females, a lower proportion with no relapses in the year prior to enrollment but similar proportions of participants reporting one or two relapses in that time frame [26,27]. Like the Slifka sample, the TOP MS sample reported fatigue, and difficulty walking as the most common current symptoms. More than half of the Slifka sample had a disability status in the first three levels of the Disease Steps, which was consistent with the TOP MS sample where the median disability scale score (EDSS) was 3.5 [26].

### Limitations

TOP MS is a prospective observational study that poses some methodological limitations as recently discussed by Marrie [28]. Lack of randomization is an inherent feature of observational studies, which can be, in part, addressed by increasing sample size. TOP MS represents the largest study of treated MS patients to be recruited for a study of compliance in the US. Observational studies sometimes lack standardized data collection, but TOP MS was designed around an electronic database that assured each patient responded to the same questions at each module and over time. Other methodological issues include possible selection bias and non-response bias. The representativeness of the enrolled TOP MS sample compared to the eligible participants from the pharmacies suggests that there has been limited selection bias within the subject pool. Non-response to the baseline surveys was 5.9% (185/3151) which compares favorably to a recent report by Jongen et al. who reported that of a sample of 163 MS patients who enrolled in a monthly electronic monitoring study 4.9% did not complete any surveys [29]. Nonresponse will be examined more completely when the full TOP MS is reported.

## Conclusions

The TOP MS Study provides a unique opportunity to examine compliance with drug use in an MS sample that appears to be representative of DMT users in the US. Due to the frequency and depth of data collection, the TOP MS database contains a wealth of information. In this report, we describe the study methodology and data collected at baseline. In future reports we will address the primary outcome of the study including longitudinal data showing change over time, outcomes and predictive variables.

## Abbreviations

DMT: Disease modifying therapy; EDSS: Expanded disability status scale; FSS: Fatigue severity scale; GA: Glatiramer acetate; ICF: Informed consent forms; IFNβ-1a IM: Interferon beta-1a, intramuscular; IFNβ-1a SC: Interferon beta-1a, subcutaneous; IFNβ-1b: Interferon beta-1b; IRB: Institutional review board; MPR: Medication possession ratio; MRI: Magnetic resonance imaging; MS: Multiple Sclerosis; MTM: Medication therapy management; NARCOMS: North American research committee on multiple sclerosis; PDQ-5: Perceived deficits questionnaire, 5 items; PHQ-9: Patient health questionnaire, 9 items; SAEs: Serious adverse events; SD: Standard deviation; SF-12v.2: Short form health survey, 12 items, version 2; TOP MS: Therapy optimization in Multiple Sclerosis; US: United States; WPAI:GH: Work productivity and activity impairment: general health.

## Competing interests

The authors make the following declaration of competing interests: PKC received personal compensation for consulting and development of educational material from Accordant, Acorda, Bayer, Biogen Idec, Genentech/Roche, Genzyme/Sanofi Aventis, Merck Serono, Mylan, Novartis, and Teva. Dr. Coyle also received research support from clinical trials sponsored by Actelion, Novartis and Opexa. BC received consulting compensation from Acorda, Astellas, Biogen-Idec, EMD-Serono, Genzyme, Sanofi-Aventis, and Teva Neuroscience. Dr. Cohen also received research support through Northwestern University from NIH, Biogen-Idec, EMD Serono, Novartis, and Hoffman La Roche and funding support through Northwestern University for a CME course from Teva Neuroscience. TL received personal compensation for consulting from Bayer, Biogen IDEC, EMD Serono, Sanofi Aventis, and Teva Neuroscience. Dr. Leist also received financial support for research activities from Bayer, EMD Serono and Teva Neuroscience. CM received personal compensation for consulting from Bayer, Biogen Idec, Eli Lilly, EMD-Serono, and Teva Neuroscience. MOB received compensation for consulting and writing from Teva Branded Products Pharmaceuticals R&D and owns stock in Teva Pharmaceuticals and other healthcare companies. MS is an employee of MedNet Solutions and receives compensation as a statistical consultant to Teva Neuroscience for this study. MT received consulting/lecture fees from Allergan, Acorda Therapeutics, Biogen Idec, EMD Serono, Genzyme, Novartis, Pfizer, and Teva Neuroscience. HZ is or has been a consultant to Acorda Therapeutics, Bayer, Biogen, EMD Serono, Genentech, and Teva Neuroscience, a member of the speakers bureau for Acorda Therapeutics, Biogen, EMD Serono and Teva Neurscience, and is a member of the advisory board for WebMD, LLC (Medscape).

## Authors’ contributions

PKC contributed to design of the study, interpretation of data, manuscript revisions for intellectual content, and final approval. BAC, contributed to design of the study, interpretation of data, manuscript revisions for intellectual content, and final approval. TL: contributed to design of the study, interpretation of data, manuscript revisions for intellectual content and final approval. CM, contributed to design of the study, manuscript review and final approval. MOB, conceived of the study, led the design of the study and interpretation of data, drafted the manuscript and made the manuscript revisions leading to final approval. MS, prepared the statistical analysis plan for TOP MS, contributed to interpretation of data, manuscript revisions for intellectual content and final approval. MT, contributed to design of the study, manuscript review and final approval. HZ, contributed to design of the study, interpretation of data, manuscript revisions for intellectual content and final approval. All authors read and approved the final manuscript.

## Pre-publication history

The pre-publication history for this paper can be accessed here:

http://www.biomedcentral.com/1471-2377/14/49/prepub
